# Neural network-based modeling of the number of microbubbles generated with four circulation factors in cardiopulmonary bypass

**DOI:** 10.1038/s41598-020-80810-3

**Published:** 2021-01-12

**Authors:** Satoshi Miyamoto, Zu Soh, Shigeyuki Okahara, Akira Furui, Taiichi Takasaki, Keijiro Katayama, Shinya Takahashi, Toshio Tsuji

**Affiliations:** 1 Department of System Cybernetics, Graduate School of Engineering, Hiroshima University, 1-4-1 Kagamiyama, Higashi, Hiroshima, Hiroshima 739-8527 Japan; 2 Department of Clinical Engineering, Hiroshima University Hospital, Hiroshima, 1-2-3 Kasumi, Minami-ku, Hiroshima, Hiroshima 734-0037 Japan; 3 Graduate School of Advanced Science and Engineering, Hiroshima University, 1-4-1 Kagamiyama, Higashi, Hiroshima, Hiroshima 739-8527 Japan; 4 Graduate School of Health Sciences, Junshin Gakuen University, 1-1-1 Chikushigaoka, Minami-ku, Fukuoka, Fukuoka 815-8510 Japan; 5 Department of Cardiovascular Surgery, Hiroshima University Hospital, 1-2-3 Kasumi, Minami-ku, Hiroshima, Hiroshima 734-0037 Japan

**Keywords:** Engineering, Biomedical engineering

## Abstract

The need for the estimation of the number of microbubbles (MBs) in cardiopulmonary bypass surgery has been recognized among surgeons to avoid postoperative neurological complications. MBs that exceed the diameter of human capillaries may cause endothelial disruption as well as microvascular obstructions that block posterior capillary blood flow. In this paper, we analyzed the relationship between the number of microbubbles generated and four circulation factors, i.e., intraoperative suction flow rate, venous reservoir level, continuous blood viscosity and perfusion flow rate in cardiopulmonary bypass, and proposed a neural-networked model to estimate the number of microbubbles with the factors. Model parameters were determined in a machine-learning manner using experimental data with bovine blood as the perfusate. The estimation accuracy of the model, assessed by tenfold cross-validation, demonstrated that the number of MBs can be estimated with a determinant coefficient *R*^2^ = 0.9328 (*p* < 0.001). A significant increase in the residual error was found when each of four factors was excluded from the contributory variables. The study demonstrated the importance of four circulation factors in the prediction of the number of MBs and its capacity to eliminate potential postsurgical complication risks.

## Introduction

The complications associated with cardiovascular surgery remain a major challenge as they can cause serious health complications^1,2^. Dementia in the elderly and severe cerebral dysfunction (developmental disorders, cognitive, language, and attention deficits) in children with congenital heart disease have been reported as the major types of complications caused by cardiopulmonary bypass^3–5^.

Advances in cardiopulmonary bypass systems have reduced complications associated with cardiovascular surgery. However, cardiopulmonary bypass surgery has been responsible for severe brain dysfunction primarily attributed to gas embolism^6–10^. Correspondingly, microbubbles (MBs) generated during the surgical field suction processes constitute a contributory factor of gas embolization. MBs are generated when blood and a large air volume flow together in the venous reservoir and are not completely defoamed^11,12^. A filter is commonly used in the venous blood reservoir and the oxygenator to protect the patient by removing inflowing air. However, it is difficult to prevent all the air that was transformed to MBs from being directed to the patient. Several studies have been reported wherein air sucked in the venous reservoir breaks down when it passes through the filter and separates into many MBs (10–20 μm)^13,14^. The MBs to be measured can be characterized in terms of quantity and size. Capillary destruction, obstructive ischemia, endothelial damage and activation of leukocytes and platelets are attributed to their numbers and sizes^7,15^. The diameters of capillaries range from 4–9 μm^16^, so that the number of microbubbles larger than this size may cause postoperative injury by being delivered. The MBs in the liquid move slowly and are delivered to the oxygenator by the negative pressure of the centrifugal pump used in cardiopulmonary bypass. The MBs delivered to the oxygenator are partially trapped, but the rest of them are sent to the patient. The number of trapped MBs depends on the type of the oxygenator. The current cardiopulmonary bypass system is equipped with a bubble sensor that can detect and warn for the presence of air bubbles with diameters ≥ 300 μm^17^, but MBs < 300 μm can potentially be sent to the patient. In this regard, current devices cannot adequately prevent the delivery of MBs.

The four factors reported to be responsible for the delivery of MBs from the venous reservoirs during cardiopulmonary bypass surgery include the surgical field suction flow rate, venous reservoir level, blood perfusion flow rate, and blood viscosity^18–22^. These factors were identified in studies that aimed to a) reduce the number of delivered MBs and b) improve the defoaming process by implemented devices with improved performances, such as oxygenators^9,23,24^. The response characteristics of MBs with respect to the contributing factors were also described^13,25^. For example, the management of the venous reservoir levels has been proposed as an approach to minimize MB delivery. Kaza et al*.*^19^ and Rubens et al*.*^20^ have respectively reported a fine adjustment pump flow method to draw blood in the surgical field and another method to separate MBs with a separate reservoir. Chung et al*.*^21^ and Souders et al*.*^22^ reported that blood viscosity is related to the buoyancy of air bubbles, such that the slow MB floating in blood. They also reported that the force of the MB delivery is related to perfusion. Collectively, these studies revealed a close relationship between each factor and MB delivery. However, a comprehensive relationship between the four factors and MB delivery has not been determined yet.

One of the difficulties encountered was that the conventional cardiopulmonary bypass system could not conduct continuous blood viscosity measurements. In response, we developed previously a method that allowed real-time monitoring of continuous blood viscosity^26^. Because the three factors (excluding blood viscosity) can be measured according to the output of the cardiopulmonary bypass, we are now able to examine the comprehensive relationship between the four factors and MBs. Blood viscosity repeatedly increases and decreases during cardiac surgery using cardiopulmonary resuscitation. Therefore, it is worth to monitor continually the viscosity and analyze its relationship with the number of MBs as well as other factors.

In this paper, we model the relationship between the number of MBs generated from venous reservoirs and the factors associated with them. First, we performed a perfusion experiment with bovine blood as the perfusate. At the same time, the values of the four factors were measured in real time, and the relationships between each factor and the number of MBs were examined. The general expression for the relationship of the number of MBs was then derived with the use of a three-layered feedforward neural network. The parameters of the model were optimized with the measured values of the four factors. The estimation accuracy of the model was validated using tenfold cross-validation. Additionally, the contribution of the continuous blood viscosity was evaluated based on the comparison of the estimation accuracies of the models constructed with and without continuous blood viscosity as one of the tested variables.

The distinctive feature of our method is the inclusion of the continuous blood viscosity that can be measured continually for the first time by the system developed by our group as a factor for the clarification of the relationship between the number of microbubbles generated and the four factors. Elucidating the relationship between the number of microbubbles generated and the factors associated with the number of MBs will enable us to identify factors that can be clinically varied and used to reduce the number of MBs. Among these factors, the suction flow rate of the operative field and the level of venous blood reservoir are factors that may be modified. Blood viscosity can be intervened in certain situations, although this has clinical limitations. This study elucidates the relationship between the number of MBs and related factors and proposes a neural network-based model that can be used during cardiopulmonary bypass surgery.

## Results

To understand the relationship between the numbers of MBs delivered from the venous reservoir during cardiopulmonary bypass surgery and the four factors (surgical field suction, venous reservoir level, blood viscosity, and perfusion flow), we performed perfusion experiments using bovine blood. In the experiment, different values of the four factors were configured to represent different perfusion conditions. At each condition, the numbers of MBs delivered from the venous reservoir were measured by a pulsed ultrasound Doppler system (model BC-100, GAMPT, Merseburg, Germany). The measurement time was 5 min at each condition. The numbers of measured MBs were represented by the median values in 1 s intervals. A correlation analysis was conducted between the four factors and the number of MBs, and results are summarized in Table 1. The values in the table show the partial correlation coefficients between each factor and the number of MBs calculated by the restricted maximum likelihood method. The results showed that the four factors moderately correlated with the number of MBs (0.4 <|*r*|≤ 0.7, *p* < 0.001). The distance to the reservoir outlet decreases as the venous reservoir level (VRL) decreases. The MBs thus immediately reach the reservoir outlet and flow out, then increasing their numbers. Therefore, a negative correlation was observed. In the case of the surgical field suction flow rate (*SFR*), the number of MBs flowing into the reservoir increases with the increase in the suction pump amount. In the case of blood viscosity (*V*), the number of MBs increases owing to the turbulent flow of blood in the vein reservoir and the decrease in the buoyancy of the incoming microbubbles as the blood viscosity increases. Blood perfusion flow rate (*Q*) increases the number of microbubbles by increasing the negative pressure that draws blood from the venous reservoir. Thus, these three factors are positively correlated with the number of microbubbles. Moreover, there were no significant correlations between all the pairs of factors. These results indicate that each factor may independently affect the occurrence and transmission of MBs.

**Table 1:** Partial correlation coefficients of tested factors (***: *p* < 0.001).

	VRL	SFR	Q	V	MB
VRL	1	0.2323	0.1665	0.2137	− 0.4978***
SFR	0.2323	1	− 0.1915	− 0.2458	0.5889***
Q	0.1665	− 0.1915	1	− 0.1232	0.4336***
V	0.2137	− 0.2458	− 0.1232	1	0.4713***
MB	− 0.4978***	0.5889***	0.4336***	0.4713***	1

Bold values indicate moderate correlation.

*VRL* venous reservoir level, *SFR* surgical field suction flow rate, *Q* blood perfusion flow, *V* blood viscosity, and MB: number of microbubbles.

**Figure 1:**
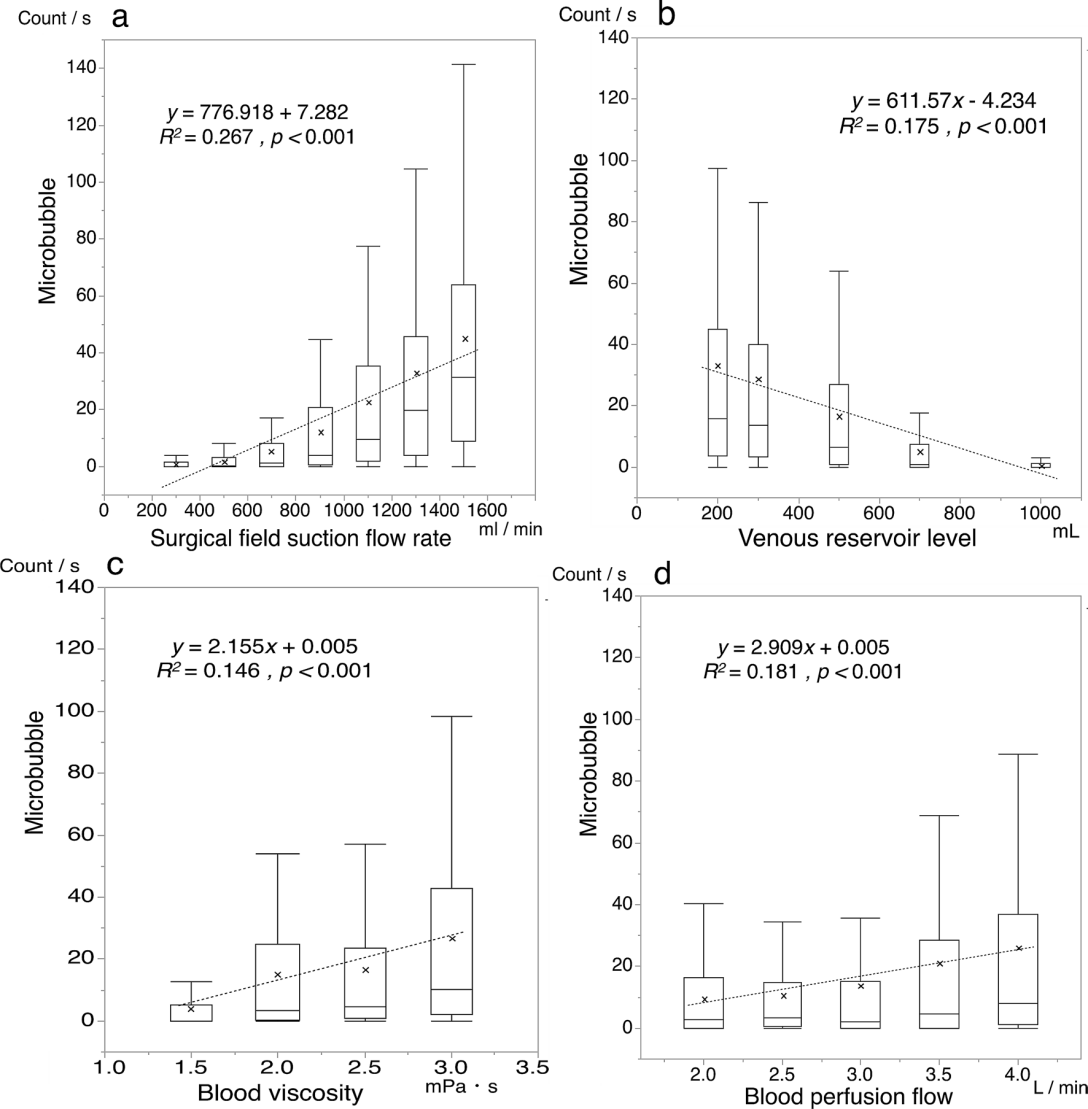
Correlation between each factor and the number of microbubbles. Dotted lines denote linear regression approximations. Plots (**a**–**d**), show the numbers of microbubbles as functions of the suction flow rate, venous reservoir level, blood viscosity, and perfusion flow rate, respectively. Cross marks denotes the average value.

Figure 1 shows plots of the number of MBs against each factor. The figure indicates that the numbers of MBs positively correlate with the suction flow rate, the blood flow rate, and the blood viscosity, and negatively correlate with the venous reservoir level. However, the dispersion is large. This result and that listed in Table 1 indicate that it is difficult to estimate the number of MBs based only on a single factor.

**Figure 2:**
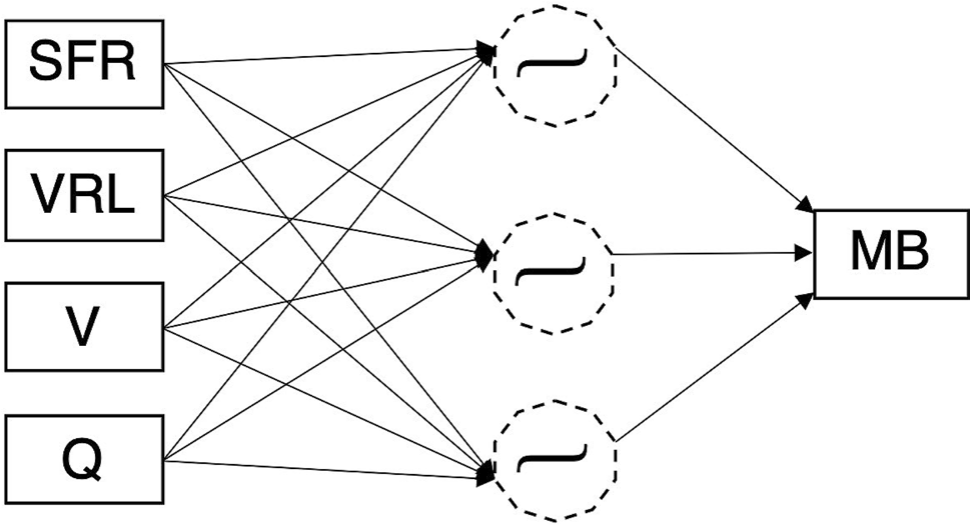
Structure of the neural network model used for the estimation of the number of microbubbles delivered from a venous reservoir.

Considering the nonlinearity in the relationship between MB and the four factors, the general expression used to show the estimated the number of MBs was derived from a three-layered feedforward neural network (see Fig. 2), and is formulated in the forms of Herein, $MB,{ }SFR, VRL, V,$ and $Q$, represent the number of MBs, surgical field suction, venous reservoir level, blood viscosity, and blood perfusion flow, respectively, and $w_{i, SFR}$, $w_{i,VRL}$, $w_{i,V}$, $w_{i, Q}$, and $w_{i}^{\left( 2 \right)}$, are the corresponding weight parameters. In addition, subscripts $i = 1,2, 3$ distinguish the neuronal units in the hidden layer, $b_{i}^{\left( 1 \right)}$ and $b^{\left( 2 \right)}$ are the bias parameters, where superscripts (1) and (2) distinguish the layers, and $y_{i}$ represents the output of the hidden layer. Further, the hyperbolic tangent in Eq. (2) was used as the activation function. The model parameters were trained and validated using tenfold cross-validation (see Methods).

1 $$ y_{i} = b_{i}^{\left( 1 \right)} + w_{i, SFR} SFR + w_{i,VRL} VRL + w_{i,V} V + w_{i, Q} Q, $$

2 $$ MB = b^{\left( 2 \right)} + \mathop \sum \limits_{i}^{H = 3} w_{i}^{\left( 2 \right)} \tanh \left( {y_{i} } \right). $$

**Figure 3:**
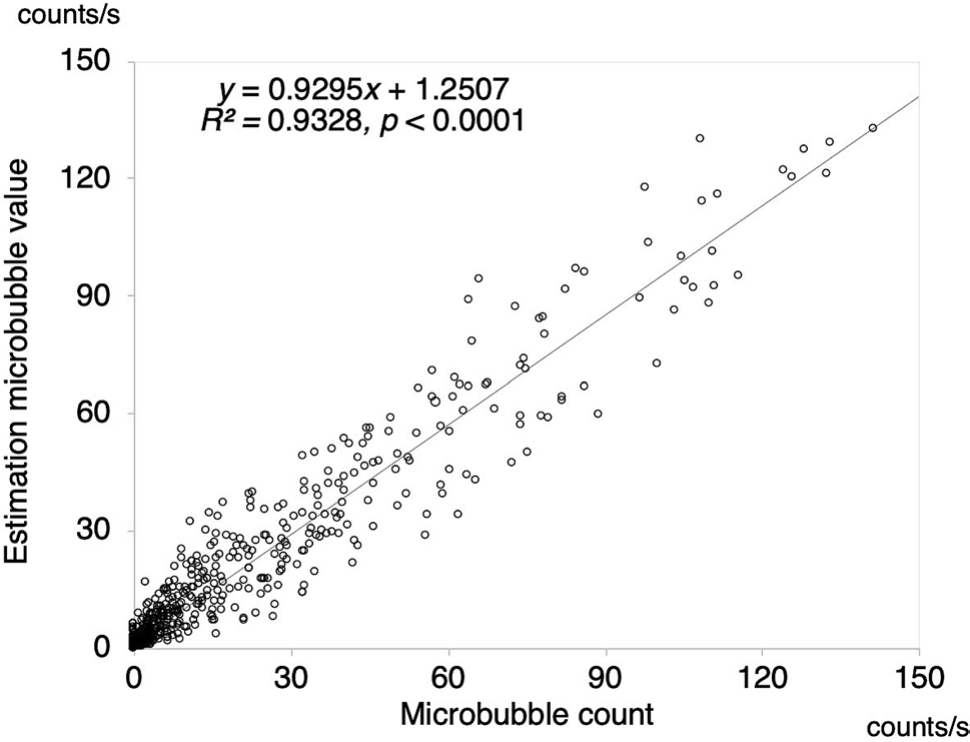
Estimation accuracy of the proposed model. The estimation number of microbubbles is plotted as a function of the microbubbles measured with a BC-100 microbubble counter.

**Figure 4:**
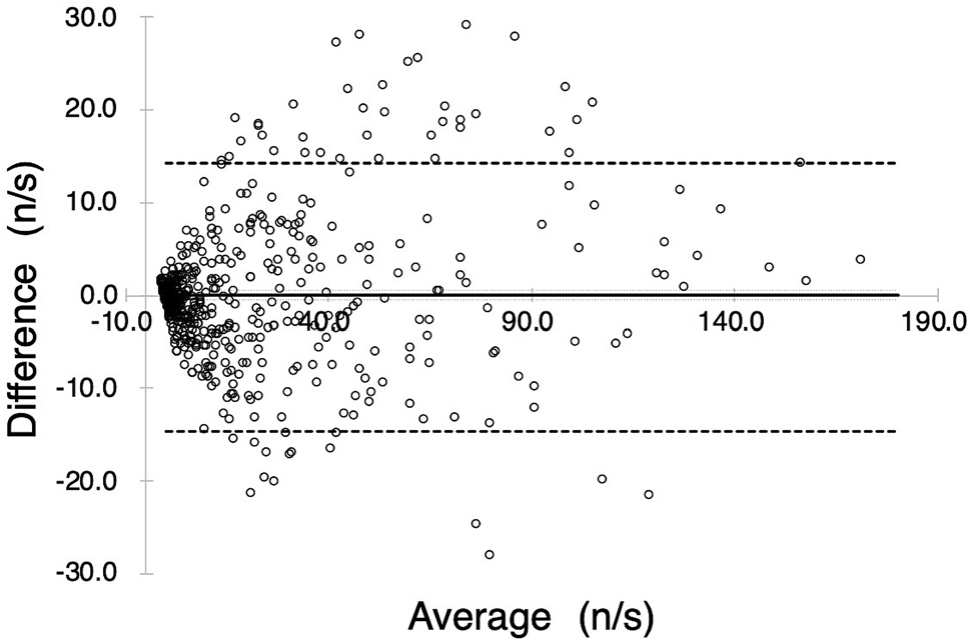
Bland–Altman plot comparing estimated and measured numbers of microbubbles. The solid line denotes the bias (mean of the difference), the large dashed line denotes the 95% limits of agreement (two standard deviations of difference), and the small dashed lines denote the 95% confidence interval for the difference between the means.

**Figure 5:**
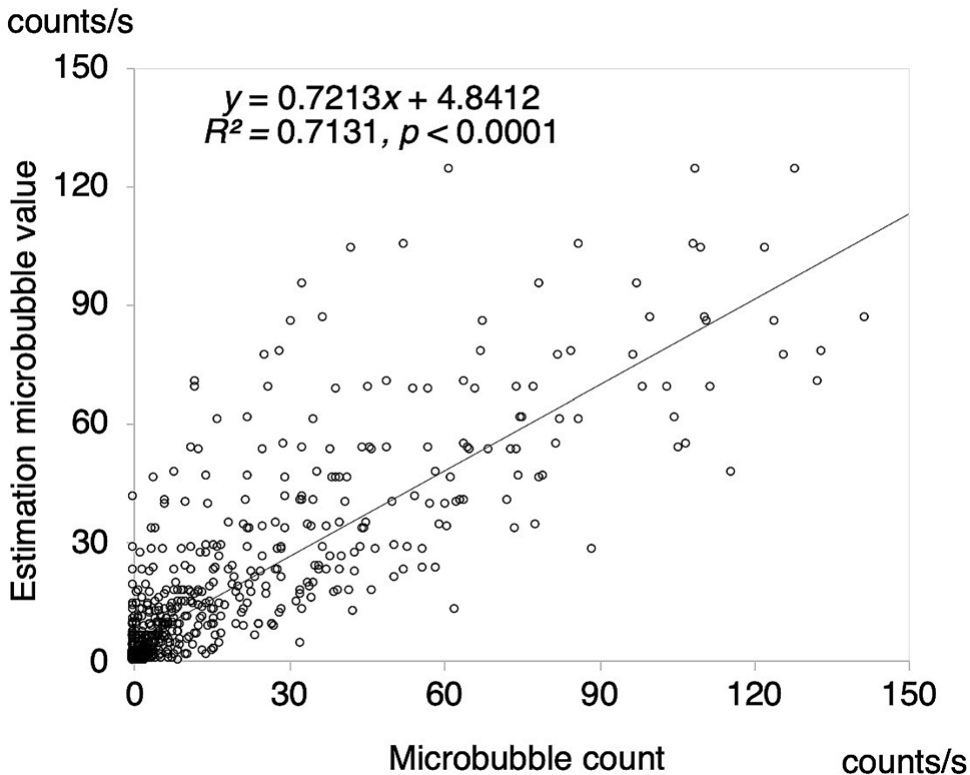
Estimation accuracy of the model in the case in which the blood viscosity factor was excluded. The estimation predicted the number of microbubbles is plotted against the number of microbubbles measured with a BC-100 microbubble counter.

**Figure 6:**
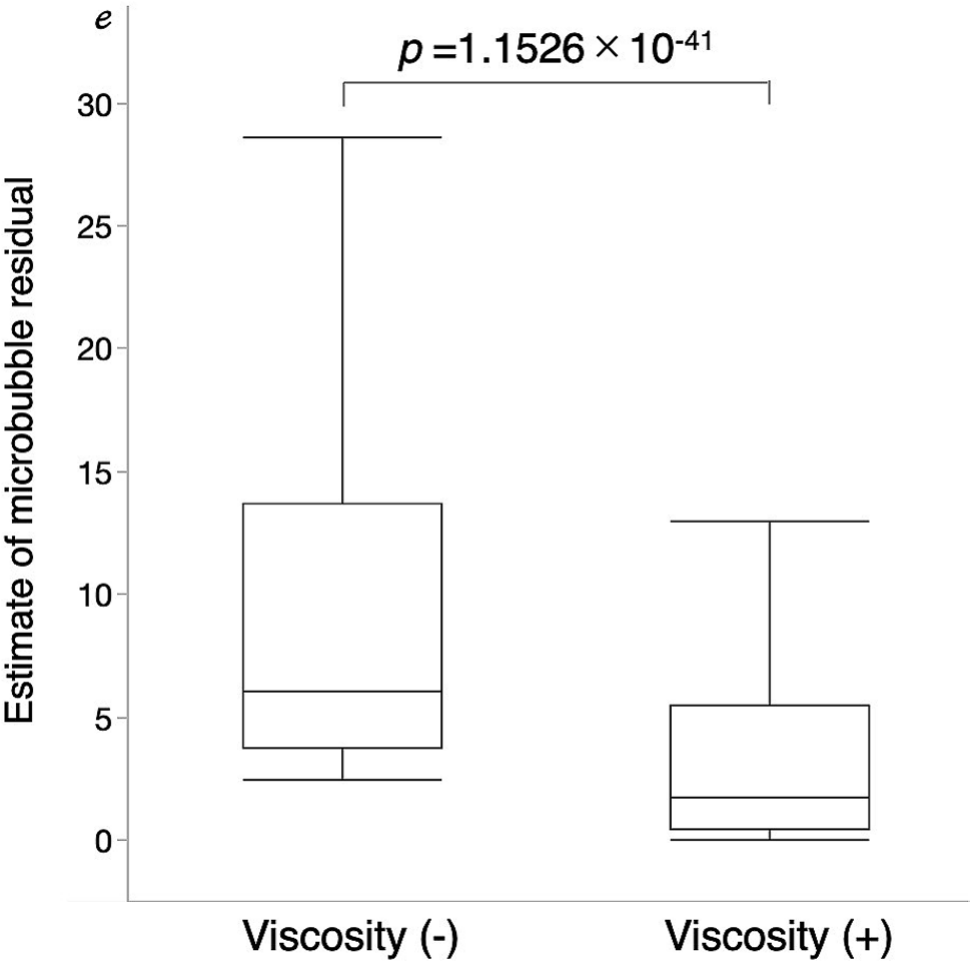
Dependency of the estimation accuracy on the blood viscosity factor. Left box plot presents the estimation residuals obtained from the model without the viscosity factor. Right box plot presents the estimation residuals of the proposed model that included the viscosity factor as one of the contributory factors. RMSE between the measured and estimated numbers of the microbubbles.

Figure 3 shows the plots of the numbers of MBs in relation to the four factors based on Eqs. (1) and (2) as a function of the measured MB (*R*^2^ = 0.9328, *p* < 0.001). This result is a plot of the values obtained from the mean of the tenfold cross-validation. Figure 4 shows a Bland–Altman analysis plot with a mean bias of − 0.06, a standard deviation of 7.35, limits of agreement (LOA) of − 14.5 to 14.2, and an error of 12.9%. These results indicate that there is no fixed or significant proportional bias (*R*^2^ = 0.1674, *p* > 0.05). Figure 5 shows the results of Eq. (1) based on the model with the blood viscosity excluded from Eq. (1). As a result, the determination coefficient between the estimation and measured MBs decreased to *R*^2^ = 0.7131. Figure 6 shows a graph with the residuals of the estimation MB and the measured MB. The residual error of the MB estimation by excluding the blood viscosity yielded a statistically significant increase (*p* = $1.1526 \times 10^{ - 41}$)*.*

**Figure 7:**
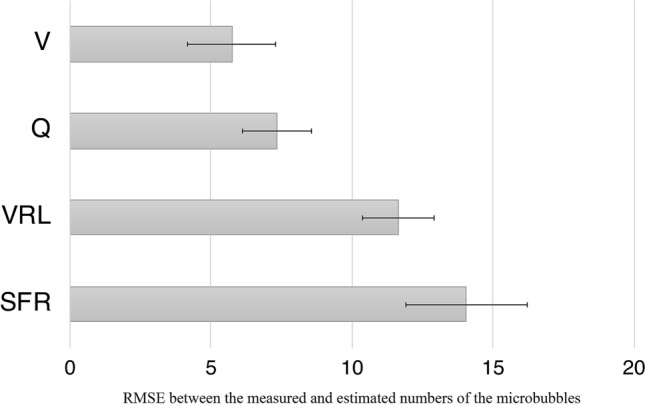
Histogram showing the evaluated the input factors to the neural network based on their permutation importance. The root mean squared error (RMSE) between the measured and estimated numbers of the microbubbles shown in the horizontal axis was used as the evaluation item: *Q* blood perfusion flow rate, *V* blood viscosity, *VRL* venous reservoir level, and SFR: surgical field suction flow rate.

It should be noted that the estimated accuracies when one of the four factors was excluded (surgical field aspiration, venous reservoir level, blood viscosity, and blood perfusion flow) from Eq. (1) were *R*^2^ = 0.3881, 0.5308, 0.7130, and 0.7742, respectively. The estimated accuracy decreased in the following order: *SFR*, *VRL*, *V*, and *Q*. This result indicates that it is effective to estimate the number of MBs using all four factors. Figure 7 shows the importance of the factors input into the neural network. The permutation importance method^27^ was used for the evaluation. The root-mean-square-error (RMSE) was used as an index, and the mean value of the RMSE was obtained by the tenfold cross-validation. The RMSEs of the surgical field suction flow rate, the venous reservoir level, the blood viscosity, the blood perfusion flow rate, respectively worsened by approximately 14.1, 11.6, 7.3 and 5.7% compared with the baseline, when random permutations were conducted on each input. The result indicates that the surgical field suction flow rate is the most effective input, and the blood perfusion flow rate was the least effective in the estimation of the number of microbubbles.

## Discussion

During cardiopulmonary bypass operations, the MBs generated in the oxygenator can cause complications when they are sent to the patient. We attempted to relate the number of MBs delivered from the venous blood reservoir with four factors^18–22^ that were the surgical field suction flow rate, the venous reservoir level, the blood viscosity and the blood perfusion flow rate. Correlation analysis indicates that the four factors were not significantly correlated with each other, but were significantly correlated with the number of MBs (see Table 1). A general expression (Eq. (1)) was then derived from a three-layered feedforward neural network, and the contributions of all the factors to the estimation accuracy were analyzed. Before using a neural network, we tested a linear model and a support vector regression model to estimate the number of microbubbles from the four factors using multiple regressions. The linear model and the support vector regression model yielded coefficients of determination of *R*^2^ = 0.5687 and *R*^2^ = 0.5595, respectively. This indicates that it is difficult to approximate the relationship between the four factors and the number of microbubbles with a linear function. Therefore, we constructed a neural network-based model of the number of microbubbles that allows us to consider the nonlinearity and the interaction between the four factors. The reservoir level was the most influential among the four tested factors. This is because a decreased reservoir level increased the distance from the location of blood suction to the liquid surface, and the blood drips from a location at an increased height generated more MBs. The recommended venous reservoir level varies from that quoted by the manufacturer, and the recommended level of the venous reservoir used in this paper was 150 ml. However, this study revealed that the number of MBs delivered from the venous reservoir increased as a function of the other three factors, even in the cases in which the reservoir level was higher than the recommended level (see Fig. 1b). The *R*^2^ for each variable was 0.5889 for the surgical field suction flow rate, 0.4978 for the venous reservoir level, 0.4713 for blood viscosity, and 0.4713 for blood perfusion flow. This indicates that there is no factor that had a dominant and exclusive impact. Accordingly, it is considered that the number of microbubbles generated was determined by a complex interaction between these four factors. We evaluated the importance of blood viscosity in relation to the number of microbubbles generated using the permutation importance method that can quantify the importance of each input feature by randomly permuting the feature values and by estimating the decrease in the RMSE^28^. The result indicated that random permutation of the blood viscosity worsened the RMSE by 7.3 points, and it was the third most important factor among the factors in microbubble generation after the surgical field suction flow rate and venous reservoir level. We found that the influence of the surgical field suction on the number of MBs was the first largest among the four factors. Meyer et al.^12^ reported that increasing the suction amount of blood in the surgical field into the venous reservoir dramatically increased MBs delivered from the venous reservoir. The deaeration process of MBs depends on buoyancy that causes them to float. In contrast, an increase in the rate of surgical field’s suction generates a drag force that acts against the buoyancy, and thus MB is pushed into the venous reservoir. Further, turbulence would also lead to an increase in the amount of generated MBs^22^. A previous study showed that the combination of the surgical field suction and reduced venous reservoir level increased turbulence effects. This could be the reason for which the surgical field suction and reduced venous reservoir level are the two most influential factors. However, the combination of these two factors was insufficient, and did not allow the estimation of the minimum amount of delivered MBs (see Fig. 1a,b).

The effects of blood viscosity were minor compared with the surgical field suction and venous reservoir level. By contrast, given that these effects are more prominent than those associated with blood perfusion flow, it can be an important factor. Based on Newtonian fluid experiments^29^ the viscosity was reported to influence MB generation and increased delivery^30^. However, the details of non-Newtonian fluids (such as blood) have been poorly documented^31,32^. Although the perfusate employed in this study was bovine blood, the reported experimental data may provide new evidence regarding the relationship between blood viscosity and the numbers of MBs generated by non-Newtonian fluids.

The defoaming process of MBs in the venous reservoir requires that the bubbles rise within the fluid and dissipate. The suction applied within the surgical field and increased blood viscosity decrease the rate of increase of the MBs^20,21^. Hence, it was considered that these factors have led to an increased number of transmitted MBs. Therefore, measuring blood viscosity in real-time is necessary to estimate the MB delivery accurately. Souders et al.^22^ reported that an increase in blood perfusion reduced the blood transit time in the venous reservoir and increased the MB delivery even though the effect was small compared with other factors. This observation was consistent in all our experimental results (see Fig. 1d).

Based on the analyzed relationship of the number of MBs against the four factors, the number of MBs can be reduced by decreasing the surgical suction pump, increasing the venous reservoir level, decreasing blood viscosity, and decreasing the blood flow rate. In cases of heavy bleeding and high surgical field suction pump flow during the operation of cardiopulmonary bypass, the number of microbubbles can be lowered by increasing the venous reservoir level by administering fluids. It should be noted that the blood viscosity is a clinically important parameter because it can be controlled by blood transfusion and fluids during surgery. The blood flow rate varies from 3.0 L/min to more than 5.0 L/min, which is usually determined depending on the patient’s body size and should be maintained at the same level during the surgery. Other factors can intervene depending on the circumstances of the operation. The safety level of the number of microbubbles, however, has not been determined so far. Thus, it is necessary to address this issue in the future. The relationship between the number of MBs and related factors was clarified, and it may be possible to intervene with the other factors to reduce the number of MBs in clinical conditions. This intervention will constitute an important implication for future clinical applications.

### Value of the research

In this study, increased estimation accuracy was achieved only when the four factors were incorporated as the contributory variables. This result indicates the importance of continuous blood viscosity that first became measurable with our previously published method^26^. Because the blood viscosity can be estimated using the outputs (differential pressure, flow rate, and temperature) commonly obtained from cardiopulmonary bypass systems, the proposed method could be easily implemented. By using the proposed system, it is possible to capture the increase in microbubbles. Given that no single factor has a prominent effect on microbubbles, the complexity of the relationship among the four factors makes it possible to predict the expected number of microbubbles. MBs sent from the venous reservoir can be reduced by adjusting one of the four factors based on the conditions during operation. Therefore, for example, the proposed model can be applied to the development of a system that can present optimal suction conditions within the surgical field.

### Limitations of this study

The safety level of the number of microbubbles has not been determined. Thus, it is necessary to address this issue in the future. The parameters are determined based on the continuous operating conditions. It is impossible to detect an increase in the number of delivered MBs owing to blood transfusion and drug administration during surgery. Additional studies are required to construct a system based on this model to identify the optimal conditions for the execution of these procedures. Also, the number of MBs may vary depending on the venous reservoir used. Finally, the test fluid used in this study was mixed with bovine blood and Ringer's solution, so that a different result may be derived when human blood is used.

## Conclusions

The number of MBs generated during cardiopulmonary bypass surgery was increased or decreased owing to the complex combinational effects of the four factors that are the intraoperative suction flow rate, the venous reservoir level, the continuous blood viscosity and the perfusion flow rate. The proposed model can estimate the number of MBs by using a neural network-based regression approach. By clarifying the relationship between the number of MBs and related factors, we were able to estimate the expected number of generated MBs with increased accuracy.

## Methods

### Test circuit

**Figure 8:**
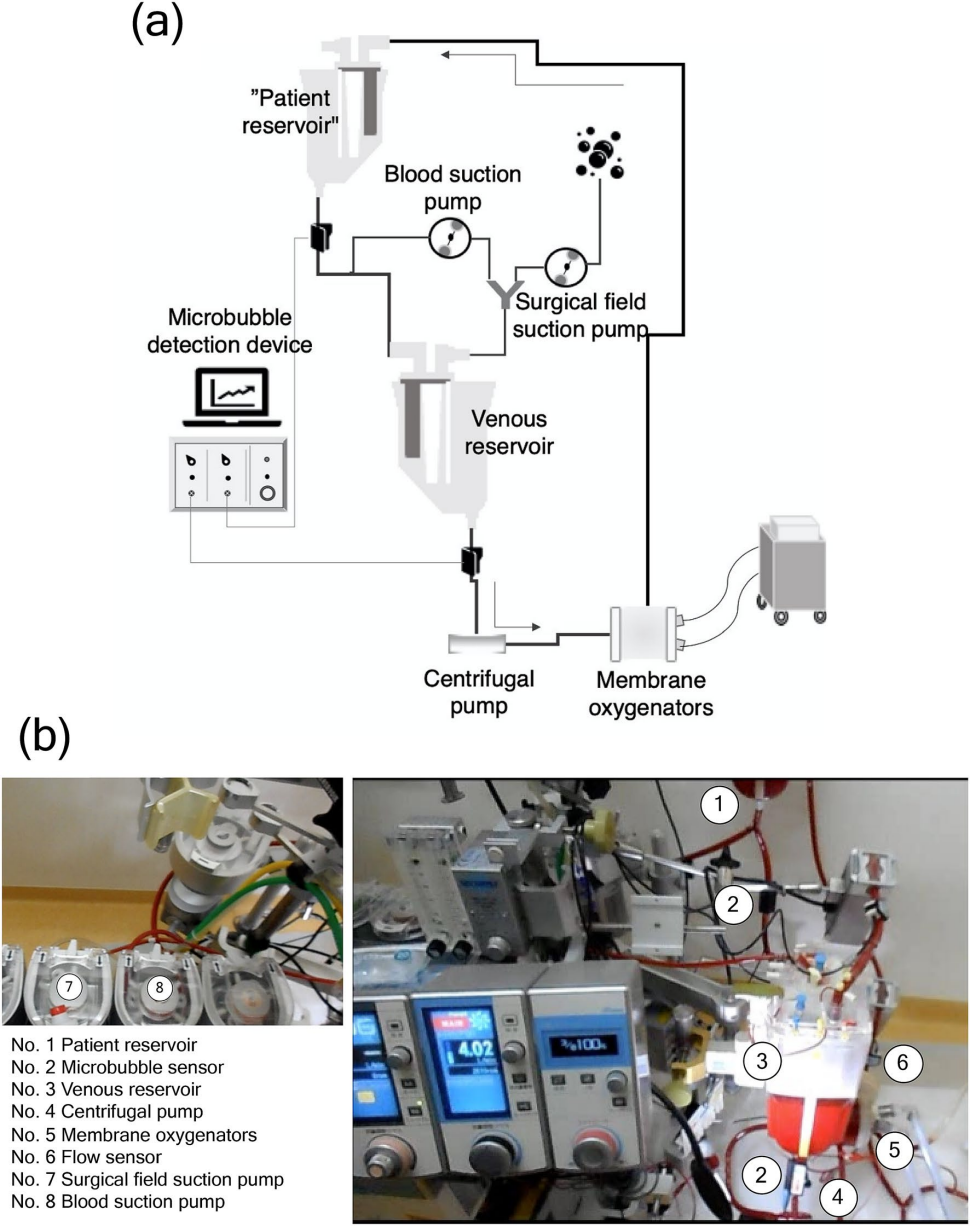
Experimental setup. (**a**) A schematic view, and (**b**) a photo. Given that the blood suction pump draws air, the pump adjusts the blood loss in the surgical field. In actual clinical practice, the surgical field (No. 7) and the blood suction pumps (No. 8) allow the estimation of the total aspirated flow rate in the operative field. Additionally, the microbubble sensor (No. 2) was installed to confirm that no air was mixed in the venous circuit from the patient reservoir, and the other microbubble sensor (No. 2) was attached to the bottom of the reservoir to measure the number of microbubbles. The blood was pumped by the centrifugal pump (No. 4), the flow rate was measured by the corresponding sensor (No. 6), and the blood was returned to the patient reservoir through the membrane oxygenators (No. 5). The patient reservoir was set up to regulate the circulating blood volume. It also contributed to the removal of microbubbles.

Figure 8 shows the test circuit. All components were new in this experiment. The patient was emulated by the hard-shell patient reservoir (Capiox venous reservoir CX-RR40, TERUMO Cardiovascular Systems Corporation, Tokyo, Japan). Owing to the fact that the recirculating circuit was constructed, the patient reservoir was filled with perfusion fluid up to a level of 1000 ml to facilitate deairing. The venous circuit was connected to the hard-shell reservoir (HSVR) via the patient reservoir. The blood was supplied to the centrifugal pump (MERA Centrifugal Pump HCF-MP23, SENKO MEDICAL INSTRUMENT, Inc., Tokyo, Japan), hollow fiber membrane oxygenator (CX-FX15E, TERUMO Cardiovascular Systems Corporation, Tokyo, Japan), and was returned to the patient reservoir. The liquid level of the HSVR was controlled by an electric regulator (HASII-RE, SENKO MEDICAL INSTRUMENT, Inc., Tokyo, Japan). Blood suction within the surgical field was dispensed from the venous circuit. In turn, this circuit was connected to the patient’s reservoir with a Y connector, and the flow rate was adjusted by a roller pump. The circuit that emulated the suction process within the surgical field used a 6 mm tube, while the airflow rate was adjusted by a roller pump. The circuit was connected with the suction blood and the connector, mixed with suction air, and returned to the HSVR.

### Experimental material

The blood reservoir volume of the HSVR was 4000 ml and included a 40 μm internal screen filter. For this reason, microaggregates, such as blood clots and fat clumps that may be sucked from the surgical field, were removed. The bubbles were structured for treatment with a polyurethane defoamer. The minimum reservoir level of the HSVR was 150 ml. In the measurement of the venous reservoir level, the hydrostatic pressure in the reservoir was continually monitored.

The membrane oxygenator used in the experiment had a built-in arterial filter that contained a screen mesh filter with a pore size of 32 μm adhered to the outside of the fiber layer. For this reason, microaggregates, such as bubbles, blood clots, and fat clumps were removed. The number of MBs was measured using a pulsed ultrasonic Doppler system (model BC-100, GAMPT, Merseburg, Germany) to detect the outflow of MBs from the HSVR. This system was set to detect MBs in the range of 10–250 μm. The probe was attached to the tube with an ultrasonic gel to eliminate air. Probes were reused in all experiments. The probe was mounted below the patient reservoir to exclude air which flowed out from the patient reservoir.

### Experimental configurations

Test fluid was prepared with 1000 ml of bovine blood and 1000 ml of Ringer's acetate solution so that the hematocrit was in the range of 24–26%. Hematocrit was not changed until the end of the experiment. The blood temperature was adjusted to 20 °C, 25 °C, 30 °C, and 35 °C, to change the blood viscosity. MB measurements began when the continuous blood viscosity became stable Experiments were performed subject to the different combinations of the experimental parameters. The rotational speed of the centrifugal pump that determined the perfusion flow rate was fixed at 2500 revolutions per minute, and reflux was performed at 4.0, 3.5, 2.5, and 2.0 ml/min based on the adjustment of the occluder. The reservoir HSVR levels were adjusted to 1000, 700, 500, 300, and 200 ml. The pump flow rates that emulated the air suction in the surgical field were 1500, 1300, 1100, 900, 700, 500, and 300 ml/min. The flow rate of the suction blood was 400 ml/min. The flow rate of 400 ml of suction blood was determined in consideration of the bleeding from the surgical field, the sampling line flow rate, the suction blood from the vent, and the hemoconcentrator flow rate. Each parameter was set within the range of the usual settings implemented in our system of the Hiroshima University Hospital. It should be noted that the flow rate of the system's surgical field suction can be set higher than that of the experimental conditions used in this study. However, the system is rarely operated in this setting, and even when it is set, it is used for a very short time. Thus, the settings for continuous operations are within the scope of this experimental method. Blood viscosity was measured continually during the experiment with the method we reported previously^26^. This system is capable of measuring blood viscosity in real time by implementing a blood viscosity estimation model based on the characteristics of the fluid that perfuses and oxygenates the membrane. The model enables calculation of the blood viscosity from the pressure and flow rate information acquired from the cardiopulmonary bypass device through a USB port. The system then displays the estimated viscosity value and its trends as well as the pressure gradient and flow rate on a computer screen to facilitate the users for monitoring the viscosity-related information. The blood viscosity was adjusted to 3.0, 2.5, 2.0, and 1.5 mPa s based on blood temperature changes. MB measurements were initially conducted at a blood viscosity of 1.5 cp and were measured at all the combinations of reservoir level, suction flow rate, and perfusion flow. After the measurements of each combination were completed, the temperature was lowered to adjust the blood viscosity. MB measurements were performed when the MB transmission volume became stable after the experimental parameters were changed. The sizes of the MBs measured with BC-100 ranged from 10 to 400 μm. The MBs were counted regardless of their sizes. The numbers of MBs were expressed as the median values of each 1 s internal. Measurements were conducted in 5 min intervals. Furthermore, CO_2_ flushing is a technique used to remove residual air in the heart. In this experiment, the gas was also blown around the air suction at 3 l/min.

### Models

First, the correlation analysis between each factor was performed by JMP14 (SAS Institute Inc. Cary, NC, USA) using the residual maximum likelihood estimation method. We then constructed a model with a multilayer neural network to enable the estimation of the numbers of MBs attributed to the four tested factors (surgical field suction, venous reservoir level, blood viscosity, and blood perfusion flow). The multilayer neural network model was implemented with JMP14. The neural network model consisted of an input, a hidden, and an output layer. Figure 2 shows the structure of the neural network employed. The model included four bias parameters and 15 weight parameters, as expressed by Eqs. (1) and (2). These parameters were trained with the gradient descent method to fit the values measured based on all combinations of the experimental configurations. The parameters of the model were validated using tenfold cross-validation where the measured data were randomly partitioned into 10 subsamples with equal sizes. The nine subsamples were used as training data, and the trained parameters were verified with the subsample remained. The model was trained using 630 data points, which corresponds to 90% of all the points, and was validated with the use of the remaining 70 points. This procedure was repeated 10 times by altering the combination of training and validation data. The accuracy of the model was examined with the correlation analysis between the estimation MB and the measured MB in addition to the residuals. To investigate the effect of blood viscosity, a model that excluded blood viscosity from the explanatory variables was also constructed. The estimation accuracy of this model was examined and compared with the estimation model that included blood viscosity as one of its variables.

## References

[1] Stoney WS, Alford WC, Burrus GR, Glassford DM, Thomas CS (1980). Air embolism and other accidents using pump oxygenators. Ann. Thorac. Surg..

[2] Gao L (2005). Postoperative cognitive dysfunction after cardiac surgery. Chest.

[3] Alston RP (2005). Pumphead–or not! Does avoiding cardiopulmonary bypass for coronary artery bypass surgery result in less brain damage?. Br. J. Anaesth..

[4] Dijk DV (2007). Octopus Study Group. Cognitive and cardiac outcomes 5 years after off-pump vs on-pump coronary artery bypass graft surgery. JAMA.

[5] Fearn SJ (2001). Cerebral injury during cardiopulmonary bypass: Emboli impair memory. J. Thorac. Cardiovasc. Surg..

[6] Murkin JM, Martzke JS, Buchan AM, Bentley C, Wong CJ (1995). A randomised study of the influence of perfusion technique and pH management strategy in 316 patients undergoing coronary artery bypass surgery. II. Neurologic and cognitive outcomes. J. Thorac. Cardiovasc. Surg..

[7] Pugsley W (1994). The impact of microemboli during cardiopulmonary bypass on neuropsychological functioning. Stroke.

[8] Groom C (2010). Microemboli from cardio- pulmonary bypass are associated with a serum marker of brain injury. J. Extra Corpor. Technol..

[9] Groom RC (2009). Detection and elimination of microemboli related to cardiopulmonary bypass. Circ. Cardiovasc. Qual. Outcomes.

[10] Abu-Omar Y, Balacumaraswami L, Pigott DW, Taggart DP (2004). Solid and gaseous cerebral microembolisation during off-pump, on-pump, and open cardiac surger y procedures. J. Thorac. Cardiovasc. Surg..

[11] Myers GJ (2007). Preventing gaseous microemboli during blood sampling and drug administration: An in vitro investigation. J Extra Corpor Technol..

[12] Svitek V, Lonsky V, Anjum F (2010). Pathophysiological aspects of cardiotomy suction usage. Perfusion.

[13] Myers, G. J., Voorhees, C., Haynes, R. & Eke, B. Post-arterial filter gaseous microemboli activity of five integral cardiotomy reservoirs during venting: an in vitro study.*J*. *Extra Corpor*. *Technol*. **41(1)**, 20–7 (2009). PMC4680219 19361028

[14] Bird JC, de Ruiter R, Courbin L, Stone HA (2010). Daughter bubble cascades produced by folding of ruptured thin films. Nature.

[15] Timme S, Eckelt N, Schmidtke E, Thomsen H (1997). Genesis and diagnostic value of leukocyte and platelet accumulations around “air bubbles” in blood after venous air embolism. Int. J. Legal Med..

[16] Lipowsky, H., McKay, C., *et al.* Microvascular mechanics: Hemodynamics of systems and pulmonary microcirculation. 13–27 (Springer Verlag NY, 1989)

[17] Kriewall TJ (1994). Safety systems in perfusion: practices, philosophy and products. Per Life..

[18] Rodriguez RA, Williams KA, Babaev A, Rubens A, Nathan HJ (2005). Effect of perfusionist technique on cerebral embolisation during cardiopulmonary bypass. Perfusion..

[19] Kaza AK (2003). Elimination of fat microemboli during cardiopulmonary bypass. Ann. Thorac. Surg..

[20] Rubens FD (2007). The cardiotomy trial: a randomised, double-blind study to assess the effect of processing of shed blood during cardiopulmonary bypass on transfusion and neurocognitive function. Circulation.

[21] Chung HK, Weber ME, Thomson J, Martin R (1981). Hydrodynamic features of pulmonary air embolism: a model study. J. Appl. Physiol..

[22] Souders JE, Doshier JB, Polissar N, Hlastala MP (1999). Spatial distribution of venous gas emboli in the lungs. J. Appl. Physiol..

[23] Nielsen, P. F., Funder, J. A., Jenson, M. O. & Nygaard, H (2008). Influence of venous reservoir level on microbubbles in cardiopulmonary bypass. Perfusion.

[24] Weitkemper HH, Oppermann B, Spilker A, Knobl HJ, Körfer R (2005). Gaseous microemboli and the influence of microporous membrane oxygenators. J. Extra Corpor. Technol..

[25] Willcox TW, Mitchell SJ (2009). Microemboli in our bypass circuits: A contemporary audit. J. Extra Corpor. Technol..

[26] Okahara S (2017). Continuous blood viscosity monitoring system for cardiopulmonary bypass applications. IEEE Trans. Biomed. Eng..

[27] Altmann A, Toloşi L, Sander O, Lengauer T (2010). Permutation importance: A corrected feature importance measure. Bioinformatics.

[28] Hou P, Jolliet O, Zhu J, Xu M (2020). Estimate ecotoxicity characterization factors for chemicals in life cycleassessment using machine learning models. Environ. Int..

[29] Clift R, Grace JR, Weber ME (1978). Bubbles, Drops, and Particles, 46–51.

[30] Mitchell SJ, Willcox T, McDougal C, Gorman DF (1996). Emboli generation by the Med tronic maxima hardshell adult venous reservoir in cardio- pulmonary bypass circuits: A preliminary report. Perfusion.

[31] Weissenborn PK, Pugh PJ (1996). Surface tension of aqueous solutions of electrolytes: relationship with oon hydration, oxygen solubility, and Bubble coalescence. J. Colloid Interfaces Sci..

[32] Vakarelskia IU (2010). Dynamic interactions between microbubbles in water. Proc. Natl. Acad. Sci..

